# Defining rational hospital catchments for non-urban areas based on travel-time

**DOI:** 10.1186/1476-072X-5-43

**Published:** 2006-10-03

**Authors:** Nadine Schuurman, Robert S Fiedler, Stefan CW Grzybowski, Darrin Grund

**Affiliations:** 1 Department of Geography, Simon Fraser University, Burnaby, Canada

## Abstract

**Background:**

Cost containment typically involves rationalizing healthcare service delivery through centralization of services to achieve economies of scale. Hospitals are frequently the chosen site of cost containment and rationalization especially in rural areas. Socio-demographic and geographic characteristics make hospital service allocation more difficult in rural and remote regions. This research presents a methodology to model rational catchments or service areas around rural hospitals – based on travel time.

**Results:**

This research employs a vector-based GIS network analysis to model catchments that better represent access to hospital-based healthcare services in British Columbia's rural and remote areas. The tool permits modelling of alternate scenarios in which access to different baskets of services (e.g. rural maternity care or ICU) are assessed. In addition, estimates of the percentage of population that is served – or not served -within specified travel times are calculated.

**Conclusion:**

The modelling tool described is useful for defining true geographical catchments around rural hospitals as well as modelling the percentage of the population served within certain time guidelines (e.g. one hour) for specific health services. It is potentially valuable to policy makers and health services allocation specialists.

## Background

The sustainability of a publicly funded universal healthcare system has become an increasing preoccupation of federal and provincial governments in Canada. In most provinces the rising cost of healthcare delivery has led to reform and restructuring to contain costs. Restructuring has generally taken the form of 'regionalization', described by Church and Barker [1] as the parallel downloading of authority and responsibility from provincial jurisdiction to regional bodies along with a similar upward movement of authority and responsibility from more local administrative bodies. Attempts at cost containment typically involve rationalizing healthcare service delivery through centralization of services to achieve economies of scale. As hospitals comprise the largest budgetary component of the public healthcare system they have been the chosen site of cost containment and rationalization [2].

Reduction of rural health services in the form of hospital downsizing or closure has consequently occurred in many Canadian jurisdictions. Smaller rural hospitals, in particular, have been targeted for closure as they are the least efficient to operate and often have limited capabilities [3]. However, the socio-demographic characteristics – and geographic distribution – of rural and remote populations present unique challenges to healthcare delivery [4]. In a mountainous jurisdiction, such as the province of British Columbia, geographic barriers impact rural settlement patterns (and transportation routes) making it difficult to maintain access to quality health services, while achieving the cost efficiencies possible in more densely populated jurisdictions [1].

Effectively planning changes to rural healthcare services require developing appropriate means to measure and assess their impact on the populations affected. Significant recent reductions in rural health services in British Columbia have occurred without appropriate means to measure the effect of the loss of local health services on the health outcomes of the surrounding population. Appropriate standards of access to rural healthcare services are currently defined by the provincial government using simple crow-fly (straight-line) distance [5], which as an approach has been shown to be less effective than more sophisticated techniques particularly in a mountainous province [6,7]. Evaluating the effectiveness of these standards is further complicated as health outcome reporting is British Columbia occurs for areas historically defined by administrative fiat and imperfectly matched to actual patterns of access to the nearest hospital service point by surface travel [8].

Healthcare administrators, planners and researchers need defined geographic areas for each rural hospital-based service based on surface travel time and the linking of this geographic catchment with the subtended resident population. This will create accurate denominator populations to link services with health outcomes [9]. The first measure of a rural hospital service's effectiveness would be the proportion of the geographic catchment served locally.

In this manuscript we present an approach that defines rural hospital catchments based on travel-time using road network data in a GIS (geographic information systems) environment. In the following sections we: 1) provide a sketch of relevant background literature; 2) describe the methodology developed to create travel-time based catchments; 3) describe the catchments produced; and 4) discuss the implications for health administrators, as well as outline subsequent research.

### Healthcare accessibility

Healthcare accessibility is multifaceted, but geographic and social barriers can thwart access to health services [10]. Geographic and social barriers to healthcare access, moreover, manifest themselves differently depending on local context and how health services are delivered. American studies often focus particular attention on lack of health insurance and inability to pay; factors that are not relevant in Canada where universal health coverage is provided. This does not preclude socioeconomic difference from impacting the accessibility of health services in Canada, but it functions in a more subtle manner – often in conjunction with geographical barriers [see [11]].

Healthcare restructuring impacts rural and remote regions differently than urban or more densely populated regions intensifying the uneven geography of health services [4]. For this reason, health planners and researchers need to pay closer attention to the impact of geography on measures of healthcare accessibility [12]. Pong and Pidblado point out that rural and remote regions typically have uneven population distributions that reduce the appropriateness of measures that use areal data, while the spatial ordering of healthcare services means that rural residents often have to 'bypass' local healthcare providers to access specialty services [see also [13]].

The spatial organization of hospitals within a region can be described in terms of an urban and functional hierarchy [14]. Spatial-interaction modelling is used to examine the spatial structure of hospital service delivery and utilization. In this view large metropolitan centres can support a wide range of services, including those that require highly specialized staff and equipment, while smaller regional centres and more remote rural communities can only support smaller primary-care hospitals. Restructuring of healthcare service delivery in Canada involves the spatial reordering of services to improve operational efficiencies [1,3]. While this necessarily involves centralization to achieve economies of scale, there are many medical conditions that require health services to be accessible within a prescribed window of time.

As a result in British Columbia standards of accessibility have been established to ensure that any changes to healthcare service delivery maintains a minimum level of access to acute care services [5]. In developing the standards the provincial government defines accessibility, safety and effectiveness, and sustainability and appropriateness as overarching principles to be maintained during the rationalization/restructuring of acute care service delivery. Similarly, assessment of acute care service delivery is expected to consider the following four factors: population/demographics; professional competence; critical mass; distance/geography. Professional competence and critical mass refer to ability to maintain the quality of healthcare services. Population/demographics and distance/geography pertain to factors affecting access and utilization of healthcare services – and are of particular interest here.

The topography and presence of physical barriers, such as mountain ranges and large water bodies, strongly affect spatial access to healthcare services suggesting that simple Euclidean distance might not be an appropriate measure of accessibility [15]. Yet, this is precisely how access to healthcare services is defined by Provincial health officials. Aerial (or crow-fly) distances are used as a proxy for travel-time thresholds mandated by the Ministries of Health Services and Health Planning [5]: emergency services (50 km = 1 hour); acute inpatient services (100 km = 2 hours); and specialty services (250 km = 4 hours). While these aerial distances are intended to approximate surface travel-time, it is unclear whether they are realistic for rural populations given that topography and the resulting population settlement patterns may make surface (road) travel much longer – and poor weather (especially during winter) may greatly impact road conditions and actual travel-times.

### Defining hospital catchments/service areas

GIS offers an array of spatial analysis methods that can be used to describe and understand the spatial dimensions of healthcare service delivery, and in particular the relationship between health outcomes and accessibility [16]. GIS has also been applied in the planning of healthcare service delivery, especially to model the optimal arrangement of healthcare facilities [17]. These analyses require, however, the ability to link healthcare services (hospitals) with potential patients; the population that might utilize their services. A catchment is a geographical area delineated around an institution or business that describes the population that utilizes its services. Normally catchments divide geographic space into contiguous regions, but in some contexts, they can overlap to reflect competition within an area between service providers [18].

Catchments in themselves do not necessarily reflect geographic proximity though they may – and perhaps should. Differences in accessibility, priority of administrative boundaries, and supply and demand for services all impact the definition of service catchments [19]. In rural British Columbia catchment definition is simplified (for acute care services) in that there is normally only one "natural" choice for patients, and it is dependent on travel-time and geographic proximity. Natural catchment definition in the province is complicated, however, by the level of services offered by proximate hospitals and perception of service quality. Health outcome reporting in British Columbia has traditionally been based on areas defined by administrative fiat that fail to adequately account for either of these factors.

There is a rather short history of rational catchment definition that has emerged in concert with growing GIS use in the fields of public health and epidemiology. Health specific catchment literature is far less numerous than literature on health accessibility. In fact, one of the few medical accounts of catchment definition and usage laments about the general lack of studies that have mapped and examined health service catchments in practice [20]. Though the situation is not quite so dire today, there are significant gaps in health literature and more generally in GIS accounts of methodologies for hospital catchment definition. GIS has discussed school catchment definition [21,22] or measured healthcare accessibility and utilization [15,23-26].

The GIS research most relevant to this research has focused on primary (or grade) school catchment definition in order to determine whether students are indeed attending the most proximate schools [see [21]]. Education and healthcare administrators require a rational way of geographically allocating populations to services provided at specific facilities. Education and healthcare planning differs in that hospitals provide differing levels of service requiring catchments that are service level specific. Schools and hospitals are both affected by perceived level of service in very different ways. Travel-time is less significant for one-time hospital visitation than regular commuting to school. Hospital catchments are therefore more likely to be affected by perception of service levels in non-catastrophic situations when distance (or travel-time) is less of a constraint. This scenario has been examined by Hanlon and Skedgel [13] with regard to cross-district utilization of general hospital care. They highlight patterns of patient travel from smaller centres to tertiary facilities in Halifax, Nova Scotia to access services that in many cases could be provided more locally.

### Methods for creating hospital catchments

There are more than a dozen possible methods for describing hospital service areas, but three predominate: Voronoi (or Thiessen) polygons, network analysis based on travel-time, and raster grid cells [27]. Voronoi polygons and network analysis are vector GIS approaches. Vector GIS refers to the use of the use of simple points, lines and polygons to represent spatial entities, whereas raster GIS uses tessellations (regularly-shaped pixels) to represent continuous surfaces [28]. Each is based on specific GIS data structures, and offers different advantages in the Canadian context.

The most common approach is to use Voronoi (or Thiessen) polygons to partition geographic space based on the locations healthcare service providers [see [23,29]]. In this method each hospital is represented as a point, and Euclidean distance is used to draw a polygon around each point such that the boundary bisects the straight line between any two points [see [28], 114]. This method presents several shortcomings – most significantly Voronoi polygons cannot incorporate facility size or capacity [30] or account for changing elevation or differing road conditions.

A more sophisticated vector GIS approach is network analysis which uses road network data in combination with information on populations and services to model resource allocations. Walsh et al. [[26], 247] describe network analysis, as employed for modelling healthcare accessibility, as

"...an approach of routing and allocating resource flows through a system connected by a set of linear features (e.g., roads and trails), where distance optimization decisions within the network are made dependent on (a) the nature of the travel conduits; (b) links between conduits; (c) location and characteristics of barriers to movement; (d) directionality of resource flows, position, and conditions of centers having specific resource capacities; and (e) node locations, where resources are deposited or collected along paths throughout the network."

Network analysis uses road network data characterized as a series of segments or links that are joined by nodes (intersections), where each link is assigned a travel cost (or impedance). Hospitals and population centroids are assigned to the nearest node. When a link or multiple links separate population and hospital nodes, link values are used to allocate population centroids to the closest hospital.

Neither Voronoi polygons nor network analysis (vector-based methods) are well suited to representing the variable conformity to catchment boundaries by a population. Hospital catchments defined using a vector approach are constrained by the reality that each polygon representing a service area is homogeneous – indicating (falsely) that the all persons are equally likely to use the hospital within the catchment. In reality, utilization of services within catchments by the resident populations is not homogeneous, but is characterized by gradations of use. At the outer boundaries of a catchment, for instance, the probability increases that a person will choose to use services in adjacent catchment. In a raster-based approach to modelling access to healthcare services, a cost surface that describes gradations of cost (measured in travel-times) between locales and proximate hospitals is developed by combining rasterized road network data with other relevant off-road network impedances; see for example the model developed by Martin et al. [[7], p.7].

While raster-based approaches offer the flexibility of representing access to services using gradations, extensive preliminary testing for this research suggests this benefit is offset by storage and computational demands. Population and road network densities are highly variable in this research's study area (British Columbia). This reality, combined with British Columbia very large geographic extent, made running a raster modelling approach at reasonable spatial resolution (50 metre pixels) problematic, especially as an eventual aim of this research is to develop a graphical user interface (GUI) that would allow various scenarios to be modelled by health professionals. Vector-based network analysis software was found to produce results comparable to those achieved by a raster approach – and sufficient for the purposes of this research – in minutes, rather than hours.

## Methods

### Developing travel-time based catchments for British Columbia's hospitals

This research employs a vector-based network analysis to model catchments that better represent access to hospital-based healthcare services in British Columbia's rural and remote areas. GIS methods have an established history of use examining the impact of distance on healthcare accessibility and utilization [17]. However, most prior research has not adequately addressed the challenges that exist in rural and remote areas where geographic barriers separate populations from healthcare services. Exceptions are Lin et al. [15], who examine the impact of distance and geographic barriers on hospitalizations in three British Columbia health regions (though still largely relying Euclidean or "crow-fly" distances), and McGregor et al. [31] who use a network analysis to assess the accuracy of Euclidean or "crow-fly" measures of access to emergency healthcare services in British Columbia's Northern Health Authority.

Martin et al. [6] examined geographical access to renal replacement therapy in the UK using three distance based approaches: crow-fly distance; road travel distance; and road travel times. Analysis revealed in a number of scenarios that simple crow-fly distance misrepresented access to health services and that depending on local context either road distance or road travel times are more effective ways to measure geographical access to services. British Columbia's rugged geography (high mountain ranges, elongated valleys, and lakes), associated inclement weather, and considerably lower population and road network densities offer a local context quite different than rural England and it is likely that these geographic differences exacerbate the errors associated with simple crow-fly distance approximations. According to Watson et al. [32] approximately 70 percent of British Columbia's total population (4,101,579 according to the 2001 Canada Census) reside on just 1.3 percent of the province's landmass. The remaining 30 percent are dispersed in a node like fashion along the highways that connect remote resource communities and non-metropolitan centres with major metropolitan areas in British Columbia (Vancouver) and Alberta (Calgary and Edmonton).

Drawing from work conducted by McGregor et al. [31] this research extends their approach using more accurate road network data to model access to specific healthcare service scenarios. ESRI's ArcGIS 9.1 [33] software was employed using the "Road Atlas of BC" dataset [34], to define travel-time based hospital catchments using the Network Analyst extension. Network Analyst provides a number of useful analytical tools that can be used to model access to services using transportation network data – including a tool for creating service areas (i.e. catchments). Creating service areas requires accurate road network data, including information about travel impedance (i.e. speed limits) and impactors (i.e. stop signs, traffic lights, etc.), and the locations of facilities. In addition, fine scale population data is required to examine the accessibility of services based on the distribution of the populations they serve.

### Road network, healthcare facility and population data

The "Road Atlas of BC" provides high quality, detailed road network data for all of British Columbia in vector GIS format – and most importantly the dataset provides segment-by-segment information on speed limits, travel impactors, and restrictions, which are needed to model realistic catchments based on travel-time calculations. The location of hospitals and other healthcare related facilities, such as health centres and ambulance stations, are provided in a point layer as part of the dataset. The hospital locations used in this analysis were queried out of the point layer based on information provided by the Interior Health Authority on their facilities, and were exported into a separate spatial layer file. Block level census data, obtained using Statistics Canada's GeoSuite 2001 software [35], was used in this analysis to link populations to healthcare services.

The "Road Atlas of BC" dataset was developed through extensive data-collection and ground-truthing and is used for a host of applications, including dispatch of emergency response services (i.e. fire, police, and ambulance). Most important for this analysis the "Road Atlas of BC" dataset contains actual speed limits collected while ground-truthing (driving) roads included in the dataset. In the context of a travel-time analysis this represents a significant improvement in data accuracy over similar road network data developed and maintained by DMTI Spatial Solutions [36] that uses estimated speeds for road segments.

Census Blocks were chosen for analysis because they are the finest-scale units for which population and dwelling counts are made available by Statistics Canada. Census Blocks contain only population and dwelling count data. No socioeconomic or demographic variables are available at the Census Block level. Though they contain no additional data, assessing geographic access to healthcare services in rural and remote areas requires fine-scale spatial data in order to link populations to the road network as accurately as possible. Dissemination Area data, the finest scale census data made available with socioeconomic and demographic variables included, offer sufficient spatial resolution only where populations are reasonably concentrated (i.e. the urban portion of rural towns and cities). Along the fringe of urban areas, or in truly rural and remote areas where populations are widely dispersed, Dissemination Area boundaries cover large geographic spaces making them unsuitable for linking populations to road networks. Census Blocks have been used because their finer-spatial resolution offers significant improvement over Dissemination Areas, but they do not completely ameliorate the problem of linking populations to road networks.

### Modelling access to hospital-based services using travel-time catchments

**Figure 1:**
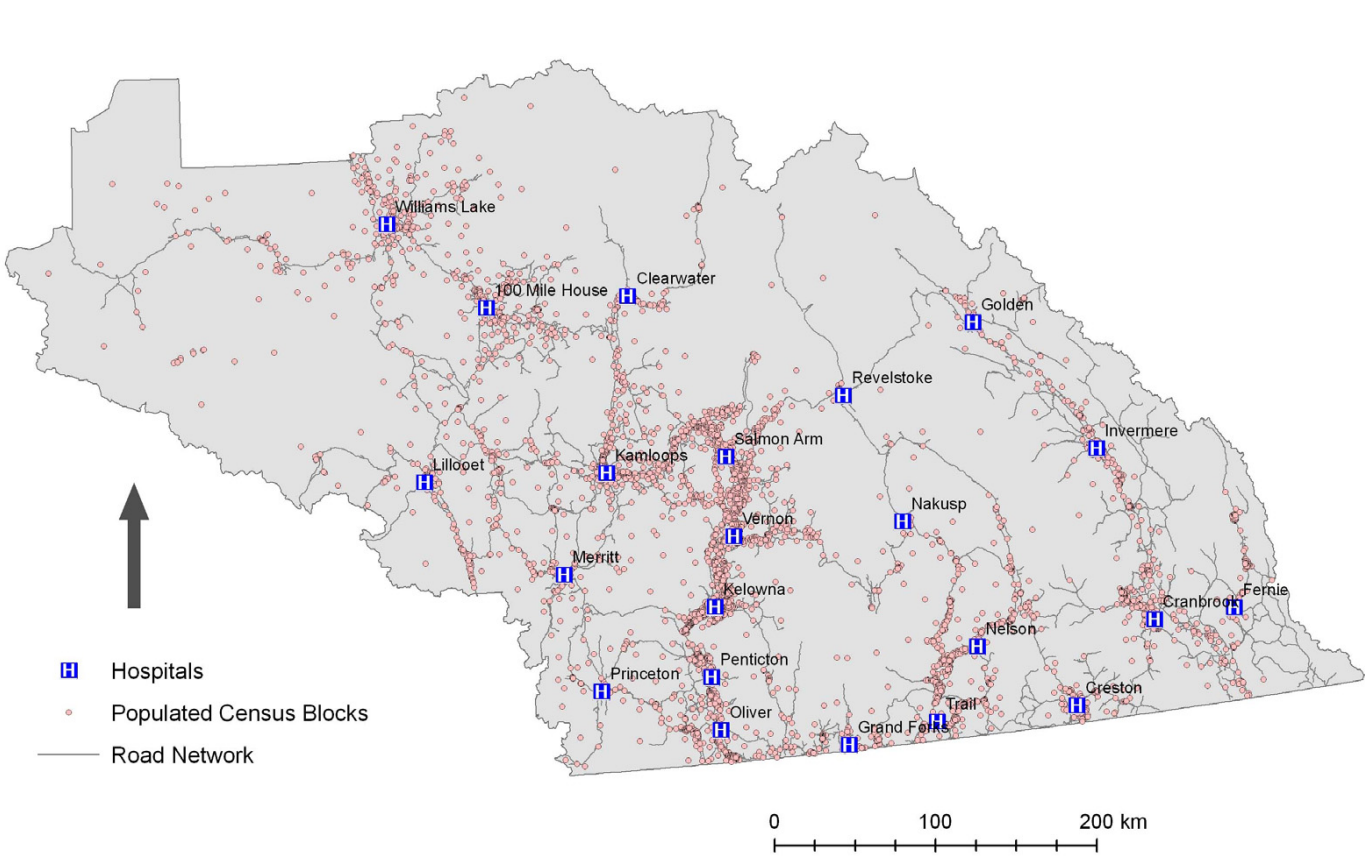
**Overview of Interior Health (IHA)**. Data Sources: GIS Innovations Ltd and Statistics Canada (2001 census).

To illustrate the utility of using travel-time based catchments three healthcare service scenarios were modelled using data for the Interior Health Authority (IHA). The IHA is responsible of healthcare planning and service delivery for a vast portion of southern British Columbia that contains approximately 655,000 people distributed in a geographically uneven manner (see figure 1). The IHA contains 22 hospitals that range in size (and capability), from tertiary hospitals in the two largest cities Kamloops and Kelowna, to small community hospitals, the smallest being Arrow Lakes Hospital in Nakusp with 6 acute-care beds (see table 1). In addition to hospitals, a number of communities are served by primary or community health centres that offer 'urgent-care.' These facilities were not considered for the purposes of analysis as they do not offer emergency services 24 hours-a-day, 7 days-a-week, nor do they provide acute inpatient-care – two key services delivered by hospitals and used by the Provincial government to define their standards of accessibility [see [5]].

**Table 1:** Access to Hospital-care Scenario Results

**Interior Health Authority**			**within 30 minutes**		**within 1 hour**	
		
		**Hospitals**	**Population**	**%**	**Population**	**%**
**Travel-Time Approach**	All Hospitals	22	575,291	87.8	623,066	95.1
	Hospitals with Critical Care & Surgeon	10	472,904	72.2	550,189	84.0
	Hospitals with Obstetrician	8	439,015	67.0	523,114	79.9
**Crow -fly Approach**	All Hospitals	22	n/a		640,077	97.7
	Hospitals with Critical Care & Surgeon	10	n/a		535,105	81.7
	Hospitals with Obstetrician	8	n/a		516,679	78.9

Note: for the crow -fly approach, 50 kilometres represents 1 hour; Total Population within the Interior Health Authority's adminstrative area is 655,044

The four scenarios modelled include:

1. Population within one hour travel-time of any hospital within the IHA (community level 1 and 2, regional, or tertiary-referral hospitals).

2. Population within one hour travel-time of any hospital offering critical care and surgical care (Intensive Care Unit and surgeon resident; 10 hospitals).

3. Population within one hour travel-time of any hospital with an obstetrician resident (the highest level of maternity care available; 8 hospitals).

4. Removal of Obstetrical service from one hospital (Royal Inland Hospital in Kamloops) and calculation of the percentage of the population affected by the closure.

To create travel-time based hospital catchments the first task was to build the road network dataset into a network dataset within ArcCatalog (the data management and browsing portion of ArcGIS 9.1). Before creating the network dataset travel cost attributes were created using line segment distance, speed limit and travel impactors. The cost to travel each way along a line segment was assigned the same cost in minutes based on the segment's length and speed limit, but additional penalty cost in minutes was calculated separately for each direction based on the presence and type of travel impactors when travelling 'to' or 'from' the segment. There is no exact method for determining the cost of travel impactors. In this analysis stop signs were assigned a 30 second penalty cost, while traffic lights 1 minute. Incorporating the cost of turns was considered, but determined to be beyond the scope of this research. The resulting network dataset was then loaded into ESRI's ArcMap (the mapping and analysis portion of ArcGIS 9.1) along with the facility location and Census Block spatial layers.

Each scenario was executed identically using the Network Analyst extension's 'create new service area' tool with only the actual facility locations considered altered between scenarios. This provides a layer that includes only the road network line segments that are within 1 hour of a hospital. Each line segment in the resulting layer also includes the facility ID of the segment's nearest hospital, which can be used to aggregate individual segments into catchments. In order to link populations to the 1 hour travel-time catchments a 2500 metre buffer was created and Census Blocks linked to it using a spatial query that selected all block centroids within it. The buffer is a means of linking denominator population to the road network. The Census Blocks selected were then exported into a separate spatial layer file and their populations summed to provide the total population within the 1 hour travel-time catchments. To compare results with the Provincial government's 1 hour "crow-fly" catchments 50 kilometre circular buffers were created for hospitals and populated using the same technique.

## Results and discussion

**Figure 2:**
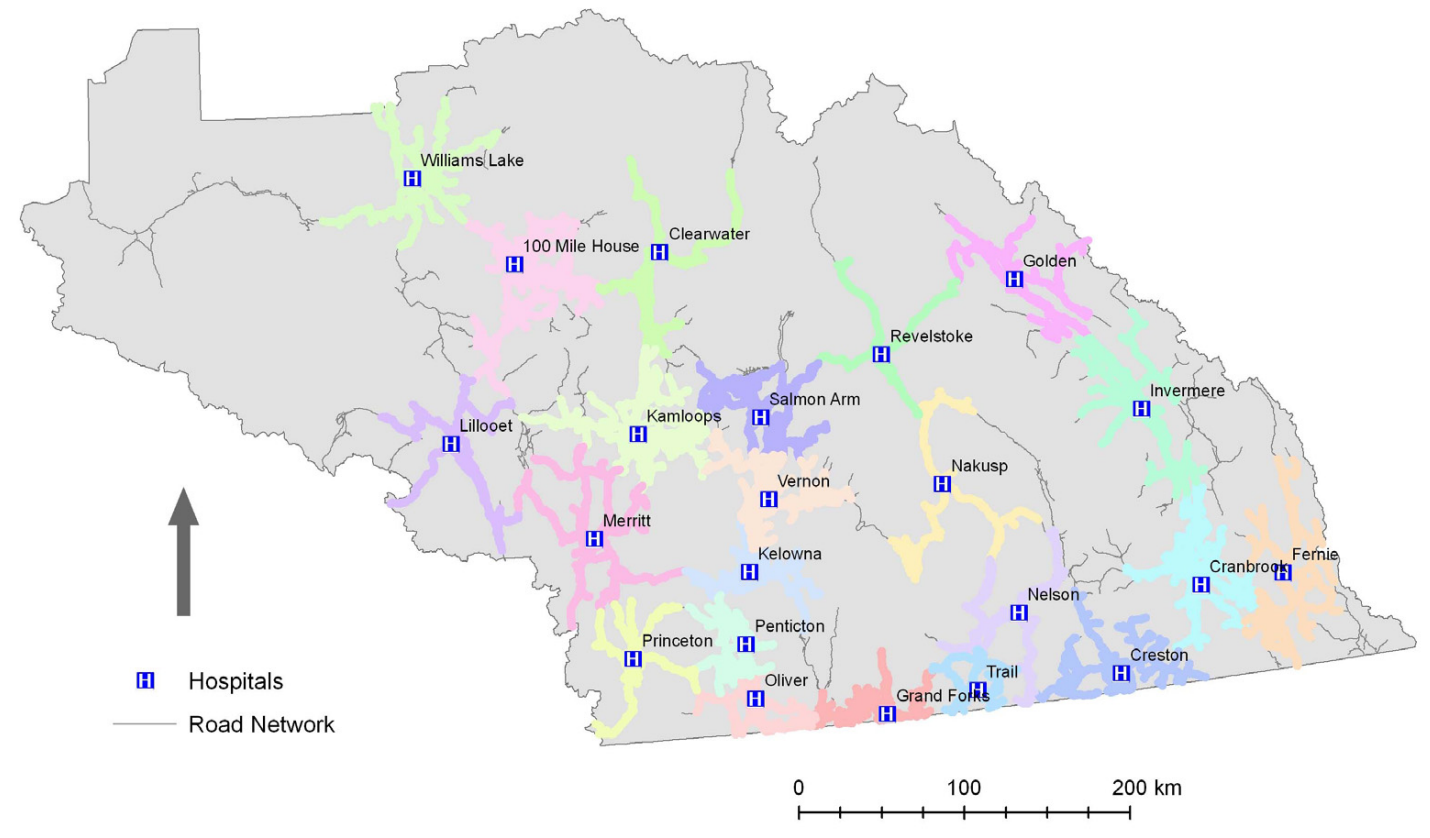
**1 hour service areas for all hospitals**. Data Source: GIS Innovations Ltd.

**Figure 3:**
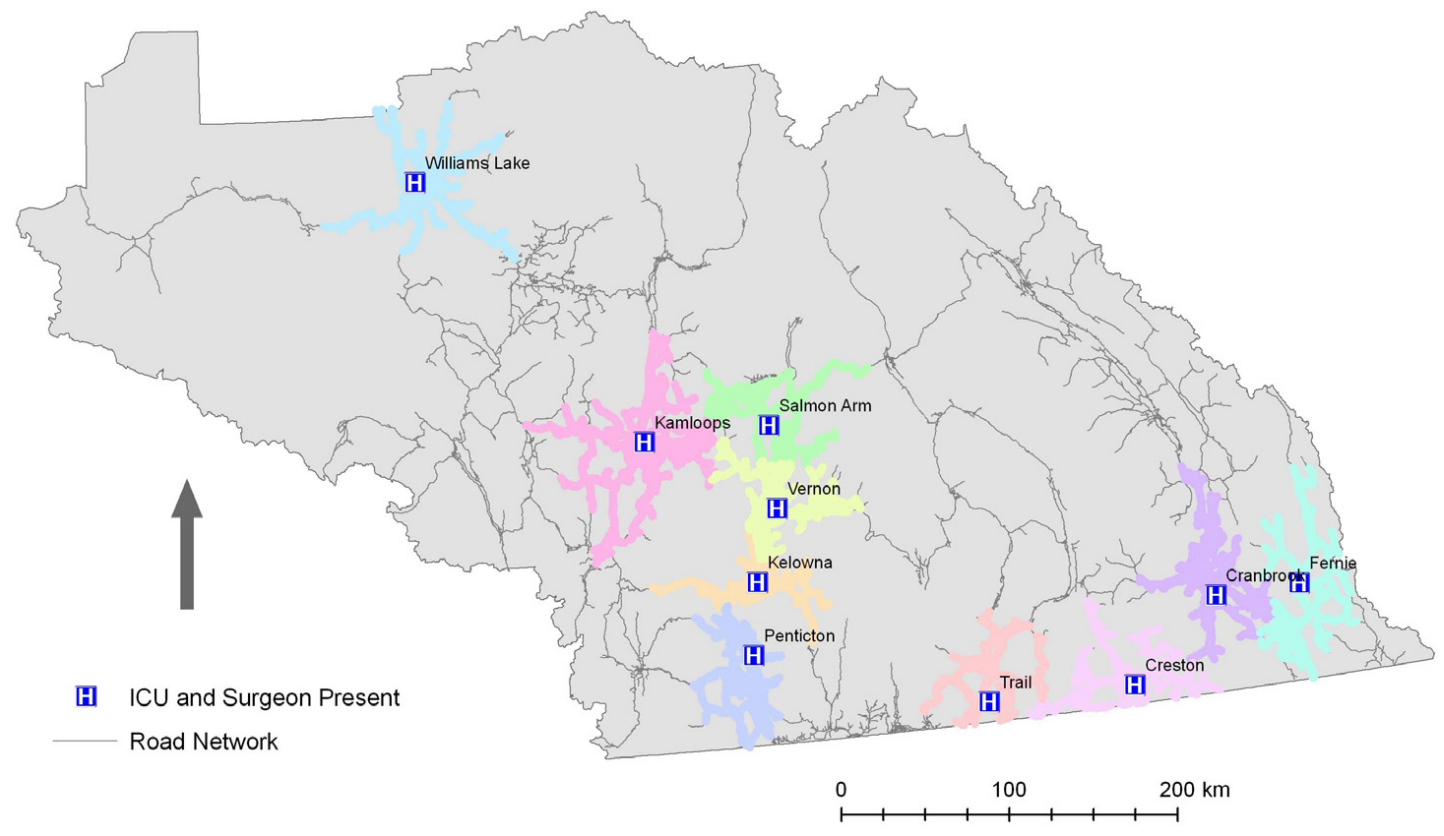
**1 hour service areas for hospitals with critical care and surgeon available**. Data Source: GIS Innovations Ltd.

**Figure 4:**
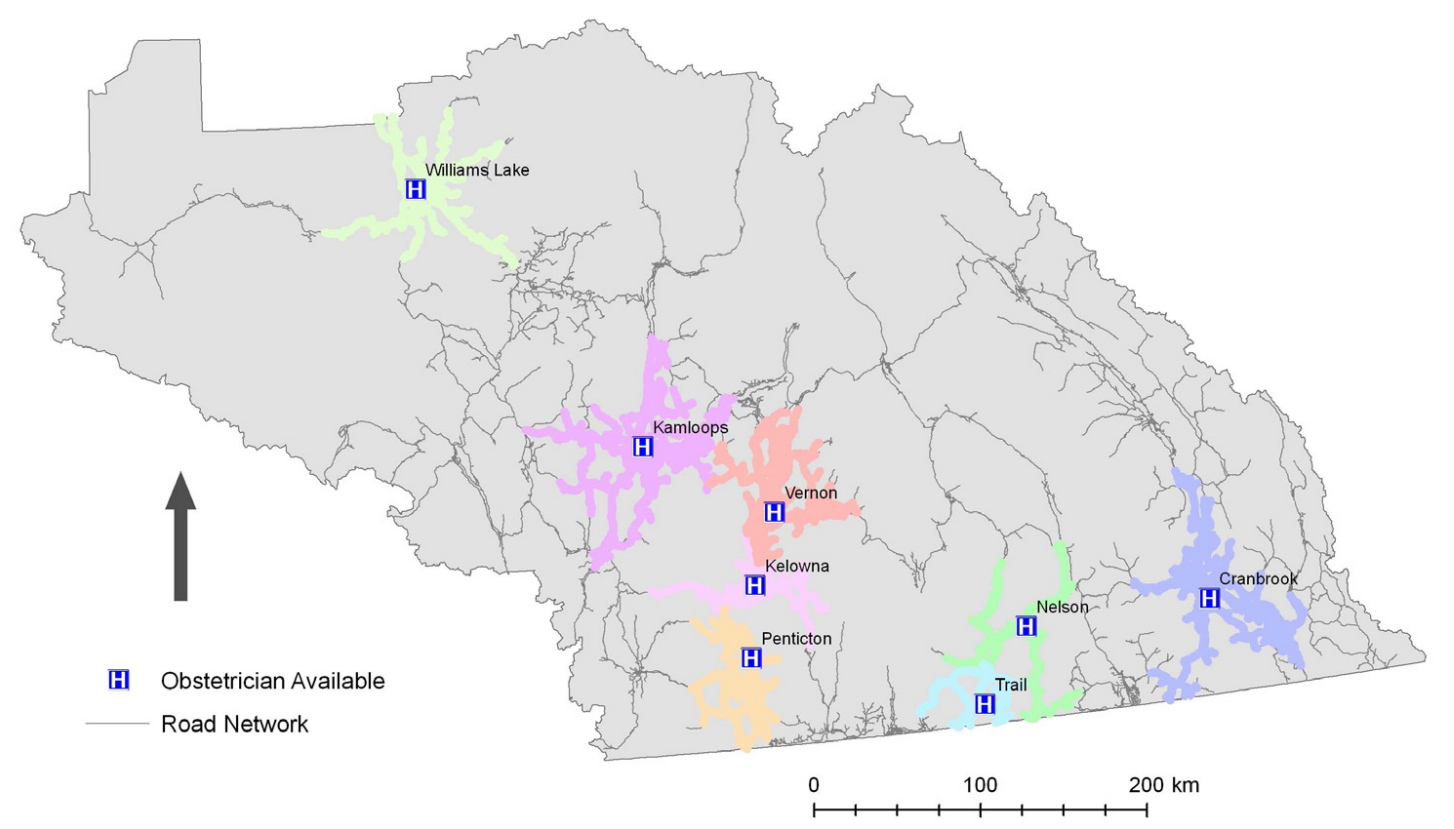
**1 hour service areas for hospitals with Obstetrician available**. Data Source: GIS Innovations Ltd.

Initial examination of the results indicate that there are few significant gaps in service as illustrated in Figure 2. This conclusion, however, does not consider the changing landscape of hospital-based health care in British Columbia. Since 2002, healthcare service delivery in British Columbia has been actively restructured. This has involved the closure or downsizing of hospitals across the province. As a result it is important to consider access to particular *services *rather than simply hospitals. Within the IHA hospitals vary considerably in terms of size and service capability, with the more numerous community hospitals significantly smaller in terms of acute-care beds and available services. The final two scenarios modelled illuminate the geographic access to more specialized services that are accessible to IHA residents. When the analysis was run for hospitals with critical and surgical care (ICU and surgeon resident), the number of hospitals falls from 22 to 10. Likewise, when hospitals offering the highest level of maternity care (i.e. Obstetrician) are considered the number of hospitals falls from 22 to 8. The reduction in geographic access – based on services levels – is illustrated by Figures 3 and 4 and Table 1.

Table 1 reveals that the difference in geographic access between the travel-time and "crow-fly" approaches are small, though clearly the populations within a 1 hour window of the hospitals considered are much lower than when all hospitals are considered (84.0 and 81.7% respectively for critical and surgical care facilities; 79.9% and 78.9% respectively for facilities with an Obstetrician resident). Interestingly the relationship between the percentage of the population within a 1 hour window of hospital care using the travel-time and crow-fly approaches is not consistent. When all hospitals in the IHA are considered the crow-fly approach finds a slightly higher percentage of the population is within the prescribed 1 hour window than the travel-time approach – the reverse of the pattern observed when specific care scenarios are examined. This pattern reveals why catchments delineated using a travel-time approach are an improvement over those developed using "crow-fly" approaches. The travel-time approach identifies populations along transportation routes that can be reasonably expected to access services within prescribed time windows more accurately. Along routes that have faster driving times – are more direct and/or have higher speed limits – further distances can be achieved within 1 hour of travel (or vice-versa for slower less-direct routes), yielding a better estimate of geographic access to hospital-based healthcare services.

Table 2 illustrates the effect of service closure on the population. In this case, Obstetrical service was hypothetically removed from Royal Inland Hospital in Kamloops. The results illustrate that were Obstetrical services not offered at that hospital, 15% of the population of the Interior Health Authority would fall into a "-99 catchment" or one without service. The method described in this paper is a means of calculating the population affected by specific service closures.

**Table 2:** Removal of Obstetric Service from Royal Inland Hosptial (Kamloops) Scenario.

Hospital Catchment	With Royal Inland Hospital		Without Royal Inland Hospital	
	
	Population	% Interior Population	Population	% Interior Population
Cariboo Memorial	26263	4.01	26263	4.01
East Kootenay Regional Hospital	34663	5.29	34663	5.29
Kelowna General Hospital	134852	20.58	134852	20.58
Kootenay Boundary Regional Hospital	25612	3.91	25612	3.91
Kootenay Lake District Hospital	28109	4.29	28109	4.29
Penticton Regional Hospital	66862	10.21	66862	10.21
Royal Inland Hospital	100156	15.29	0	0.00
Vernon Jubilee Hospital	99760	15.23	99888	15.25

-99 Catchment (population not served)	138843	21.19	238871	36.46

Total Interior Population	655120	100.00	655120	100.00

Note: 15% of the population in the IHA are presently served by Obstetrical services at the Royal Inland Hospital. When that service is removed in this scenario, the -99 catchment – representing the population not served within one hour travel time – increases by 15%.

Specific scenario modeling should not obscure the trend that, when more specialized health services are considered, a clear difference in geographic access emerges within the IHA. The Okanagan area of the IHA, which includes cities of Vernon, Kelowna and Penticton, appears to be well serviced, while geographic access is less generous in the Kootenay region – especially in the East Kootenays. Given the small and relatively remote populations that characterize the East Kootenay area this finding is not unexpected. Following the logic of regionalization reform that advocates the centralization of services in order to achieve economies of scale, one would expect to find hospital-based healthcare services to exhibit a hierarchical spatial organization. Modelling the spatial distribution of health services in a GIS environment offers an efficient and flexible way to examine patterns of geographic access, but for results to be of practical use policy-makers must also consider the medical and political aspects of healthcare service delivery [37].

Healthcare restructuring in other Canadian jurisdictions has revealed the closure of hospitals does not automatically result in poorer health outcomes or unacceptable access to inpatient services for rural populations [38], but assessing the impact of hospital closure on small rural communities is more complex. James [39] points out that the impact of hospital closures goes beyond simply changing rural residents' geographic access to hospital-based services; it also affects how rural residents view the long-term sustainability of their communities. This is an aspect of healthcare restructuring that evades easy incorporation in GIS models of healthcare accessibility and is beyond the scope of the approach presented here. It does, however, reveals an important limitation of catchments or service boundaries delimited by time and/or distance and suggests a valuable direction for future research.

## Conclusion

There is increasing pressure to rationalize location and delivery of health services whilst continuing to provide comprehensive care to rural residents. This research has demonstrated the utility of modelling travel time catchments for rural hospitals. Modelling catchment scenarios can be used to illustrate a) the percentage of denominator population that is outside of a one, two or four hour catchment; b) variations in service coverage when specific groupings of services are considered (e.g. rural maternity or critical care); and c) changes in catchment definition and population served when a hospital and/or service is removed or added.

This tool is poised to rationalize health services planning for non-urban areas. To that end, research is underway to create a Graphical User Interface (GUI) that will permit hospital administrators to model different scenarios of service provision.

## Competing interests

The author(s) declare that they have no competing interests.

## Authors' contributions

Nadine Schuurman participated in the conceptualization of the study, designed the methodology, secured the data and supervised the analysis.

Robert Fiedler developed and ran the network analysis.

Stefan Grzybowski demonstrated the need for the analysis and participated in the conceptualization of the study.

Darrin Grund designed and tested raster implementations of the methodology.
